# Contextual Information Modulates Pupil Size in Autistic Children

**DOI:** 10.3389/fnins.2022.752871

**Published:** 2022-04-01

**Authors:** Chiara Tortelli, Antonella Pomè, Marco Turi, Roberta Igliozzi, David C. Burr, Paola Binda

**Affiliations:** ^1^Department of Surgical, Medical, Molecular and Critical Area Pathology, University of Pisa, Pisa, Italy; ^2^Department of Neuroscience, Psychology, Pharmacology and Child Health, University of Firenze, Firenze, Italy; ^3^Fondazione Stella Maris Mediterraneo, Matera, Italy; ^4^IRCCS Stella Maris Foundation, Pisa, Italy; ^5^Department of Translational Research on New Technologies in Medicine and Surgery, University of Pisa, Pisa, Italy

**Keywords:** pupillary light reflex, contextual effect, pupillometry, autism, individual differences

## Abstract

Recent Bayesian models suggest that perception is more “data-driven” and less dependent on contextual information in autistic individuals than others. However, experimental tests of this hypothesis have given mixed results, possibly due to the lack of objectivity of the self-report methods typically employed. Here we introduce an objective no-report paradigm based on pupillometry to assess the processing of contextual information in autistic children, together with a comparison clinical group. After validating in neurotypical adults a child-friendly pupillometric paradigm, in which we embedded test images within an animation movie that participants watched passively, we compared pupillary response to images of the sun and meaningless control images in children with autism vs. age- and IQ-matched children presenting developmental disorders unrelated to the autistic spectrum. Both clinical groups showed stronger pupillary constriction for the sun images compared with control images, like the neurotypical adults. However, there was no detectable difference between autistic children and the comparison group, despite a significant difference in pupillary light responses, which were enhanced in the autistic group. Our report introduces an objective technique for studying perception in clinical samples and children. The lack of statistically significant group differences in our tests suggests that autistic children and the comparison group do not show large differences in perception of these stimuli. This opens the way to further studies testing contextual processing at other levels of perception.

## Background

Although atypical perception is not a diagnostic criterion for Autism Spectrum Disorders, there is growing evidence that autism is associated with different perceptual styles (Hermelin and O’Connor, 1970; Behrmann et al., 2006; Simmons et al., 2009; Hadad et al., 2017). The idiosyncrasies in visual perception include a preference for local over global perceptual features (Shah and Frith, 1983; Jolliffe and Baron-Cohen, 1997; Chouinard et al., 2016), reduced susceptibility to visual illusions (Happe, 1996), and deficits in the processing of contextual information for social cues (Ames and Jarrold, 2009), face processing (Teunisse and de Gelder, 2003) and perceptual grouping (Brosnan et al., 2004). Several recent theories link autistic perception with Bayesian models of sensory integration (Pellicano and Burr, 2012; Friston et al., 2013; van Boxtel and Lu, 2013; Lawson et al., 2014; Sinha et al., 2014; Van de Cruys et al., 2014; Rosenberg et al., 2015; Palmer et al., 2017). The fundamental idea is that perception is more “data-driven” in autistics than in neurotypicals, less dependent on contextual information that is known to strongly influence perception under many circumstances.

Experimental tests of susceptibility to illusions have produced mixed results. Some behavioral studies found reduced susceptibility to illusions in autistic individuals (Happe, 1996; Bolte et al., 2007; Mitchell et al., 2010), but others failed to detect significant differences in the strength of illusory effects between autistic and controls (Ropar and Mitchell, 1999, 2001; Hoy et al., 2004; Milne and Scope, 2008; Manning et al., 2017). For example, using the method-of-adjustment (where participants adjust one stimulus until it is perceptually identical to another), Ropar and Mitchell (1999) found that autistic children are generally similarly susceptible to illusions as children from a range of comparison groups, including individuals with moderate learning difficulties and typically developing children and adults. Similarly, autistic participants have been reported to perceive the orientation of low-level stimuli in a qualitatively similar manner as control participants, with no evidence of superior processing in the precision or accuracy of orientation perception (Brock et al., 2011; Shafai et al., 2015).

Several confounding factors might explain inconsistencies in earlier work. Perseverative behaviors, anxiety, and understanding of task instructions are difficult to control for. Compounding this problem, many studies used behavioral paradigms that may be influenced by strategies in reporting what they perceive. Previous studies assessing visual illusions in autism have confounded sensitivity to an illusion with the subjective criterion for reporting the illusion. Therefore, group differences in illusion susceptibility estimates may reflect differences in decision criteria or bias, with no underlying differences in perception [a possibility that is particularly likely when groups differ in cognitive and affective factors (Skottun and Skoyles, 2014)].

For these and other reasons, it would be useful to measure objective and quantitative indices of perception and perceptual styles. Recent work suggests that pupillometry may serve this purpose. Pupillary constriction in response to light increment is probably the simplest visually evoked response. However, higher order visual processes such as attention (Binda et al., 2013a,^2014^; Binda and Gamlin, 2017; Ebitz and Moore, 2017), visual awareness (Einhauser et al., 2008; Fahle et al., 2011; Naber et al., 2011; Kimura et al., 2014; Sperandio et al., 2018), mental imagery (Laeng and Sulutvedt, 2014) and brightness illusions (Laeng and Endestad, 2012; Zavagno et al., 2017) can also contribute to determining pupil size (Binda and Murray, 2015; Mathot and Van der Stigchel, 2015). For example, contextual cues associated with high light levels, such as an image of the sun, cause pupillary constriction compared with more neutral luminance-matched images (Binda et al., 2013b; Naber and Nakayama, 2013; Sperandio et al., 2018; Castellotti et al., 2020). These results show that pupillary constriction typically reflects the interpretation of light in a scene, not just the amount of physical energy entering the eye, suggesting that pupillary responses can be informative about an individual’s perception. Indeed, pupillometry has been shown to track inter-individual differences in perception, and it may even be more reliable than behavioral measures and other physiological responses (Turi et al., 2018; Pome et al., 2020; Tortelli et al., 2020, 2021).

In the present study, we used a no-report pupillometry paradigm to assess processing of contextual information in autistic children and a comparison group. Specifically, we tested whether a pictorial representation of the sun would lead to pupillary constriction when compared with other luminance-matched images, using the same stimuli as in Binda et al. (2013b). After validating a novel child-friendly paradigm in a group of neurotypical adults (Experiment 1), we measured pupillary response to images of the sun and to meaningless phase-scrambled images matched in luminance and contrast in two groups of children: one with autism diagnosis and the other presenting developmental disorders unrelated to the autistic spectrum, matched for age and IQ (Experiment 2). The results show that groups did not differ, and that both had stronger pupillary constrictions for the sun compared with control images, similar to the neurotypical adults. Our observations do not support the hypothesis of reduced use of contextual information in autism.

## Materials and Methods

### Participants

Experiment 1 was conducted on 40 neurotypical adults (32 females; age mean and SD: 29.8 and 1.69 years), with no diagnosed neurological condition. For Experiment 2, we recruited 41 children with developmental disorders, including 18 autistics (3 females; age 6.2–15.6 years; mean and SD: 11.0 and 0.6 years). Note that we often use the wording “autistic” throughout the paper, aligning with the preference for identity-first language expressed by the autistic community (Kenny et al., 2016). The comparison group comprised 23 children (7 females; age 6.8–15.5 years; mean and SD: 10.5 and 0.6 years) diagnosed with disorders considered to be outside the autism spectrum, specifically: learning disabilities (LD) (*n* = 7), developmental language disorder (DLD) (*n* = 7), behavioral disorder (BD) (*n* = 3), or attention deficit hyperactivity disorder (ADHD) (*n* = 5).

Children in the autistic group had received a diagnosis of autism according to DSM-5 criteria (APA, 2013), or of autistic disorder, Asperger disorder, and pervasive developmental disorder-not otherwise specified according to DSM-IV-TR criteria (American Psychiatric Association, 2000; Table 1). In all cases, the diagnosis had been made prior to admission in the study, by a multidisciplinary team that included a senior child psychiatrist and an experienced clinically trained research child psychologist. The autistic and comparison groups were matched by chronological age [two-sample *t*-test on age in years: *t*_(39)_ = 0.5, *p* = 0.60, lgBF = –0.5] and Performance IQ [*t*_(39)_ = 0.2, *p* = 0.81, lgBF = –1.04], as measured by standardized tests (Leiter International Performance Scale-Revised or Leiter-R (Roid and Miller, 1997); Wechsler Preschool and Primary Scale of Intelligence WPPSI, Italian version (Wechsler, 1989); Wechsler Intelligence Scale for Children (O’Donnell, 2009), chosen for each participant based on their varying levels of verbal functioning. All children had a Performance IQ score above 70 and were thus considered “cognitively able.” No child in either group had additional medical or developmental conditions, as reported by parents, and no child was on medication at the time of the study (Table 2).

**TABLE 1:** Mean (standard deviations) in each group of participants; the last column gives the comparison between the two groups of children.

	Adults	Autistics	Comparisons	A vs. C
Gender (F:M)	32:8	3:15	7:15	X^2^ = 1.2, *p* = 0.27
Age	29.8 (1.6)	10.9 (2.3)	10.5 (2.9)	*t*_(39)_ = 0.5, *p* = 0.60
Performance IQ	–	99.0 (17.0)	100.4 (14.1)	*t*_(39)_ = 0.3, *p* = 0.78
Ados-2 total score	–	12.2 (3.3)	–	
AQ total score	14.2 (8.01)	27.6 (7.8)	18.3 (7.5)	*t*_(39)_ = 3.8, *p* < 0.0001

**TABLE 2:** Mean (standard deviations) in each subgroup of the comparison group of non-autistic participants.

Comparison group:	Learning disabilities	Developmental language disorder	Behavioral disorder	ADHD
Gender (F:M)	2:5	2:5	1:2	2:4
Age	11.9 (2.56)	7.97 (1.4)	9.4 (2.56)	12.3 (2.93)
Performance IQ	100.5 (13.81)	108.7 (12.33)	91.7 (12.74)	99.0 (16.11)

Using G*Power (Faul et al., 2007), we computed the sensitivity of our tests given the sample size, a set power of 80% and a type I error probability of 5%. This indicated that the smallest between-group difference we would be able to detect was 0.9 as measured with Cohen’s d metrics, a large effect size.

All participants had normal or corrected-to-normal visual acuity. Experimental procedures were approved by the regional ethics committee *Comitato Etico Pediatrico Regionale—Azienda Ospedaliero-Universitaria Meyer—Firenze (FI)* and are in accordance with the declaration of Helsinki; participants (and their legal guardian, where appropriate) gave written informed consent.

### AQ Score

All neurotypical adult participants filled out an on-line or paper version of the Autism-spectrum Quotient questionnaire, using the validated Italian version (Baron-Cohen et al., 2001; Ruta et al., 2012). The test comprises 50 items. Responses are made on a 4-point Likert scale: “Strongly agree,” “slightly agree,” “slightly disagree,” and “strongly disagree.” Items were scored as described in the original paper (Baron-Cohen et al., 2001): 1 when the participant’s response was characteristic of autism (slightly or strongly), 0 otherwise. Total scores ranged between 0 and 50, with higher scores indicating higher degrees of autistic traits. AQ scores for children are parent-reported, and were collected using the age-appropriate form (Auyeung et al., 2008). For one child participant the AQ score was not collected (the parent filled out only part of the questionnaire and could not be re-contacted to complete the task).

### Stimuli and Procedure

The experiment was conducted in a dark room with no illumination other than the display screen. For adults (Experiment 1) the display was a CRT (Cathode-ray tube) monitor (40 × 30 cm, Barco Calibrator with resolution 1,024 × 768; maximum-minimum luminance 53–0.1 cd/m^2^). Children (Experiment 2) were tested with a more portable device (53 × 32.8 cm LCD color monitor Acer, with resolution 1,920 × 1,080; maximum-minimum luminance 110–0.1 cd/m^2^). In both cases, the screen was placed 57 cm from the participant, whose head was stabilized by chin rest. Visual stimuli were generated in Matlab (Mathworks) using the Psychophysics Toolbox (Brainard, 1997). Total testing time (for both adults and children) was about 30 min, including the time for initial adjustment of the apparatus to match each participant’s eye-level.

**FIGURE 1:**
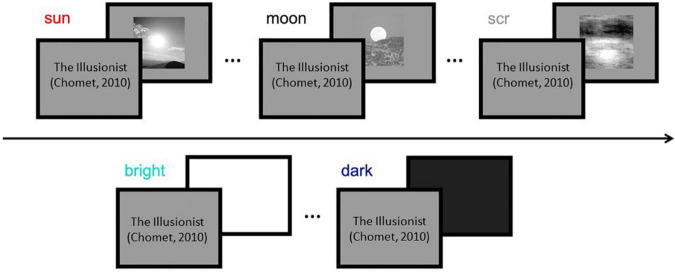
Schematics of the experimental stimuli and procedure. **Top:** images (sun, moon, and meaningless control images) were presented for 1s each in random order, embedded within an animation movie (Chomet, 2010; for copyright reasons the frames of the cartoon are replaced by title and author of the movie in this Figure). Note: all tested stimuli were achromatic; the movie (in color) merely filled the inter-stimulus intervals and was common to all conditions. **Bottom:** the same protocol was used for the presentation of full-screen maximum or minimum luminance squares, for testing the pupillary light or dark response.

During experimental sessions participants observed a clip extracted from an animation movie (Chomet, 2010), displayed at screen center within a window of 17 × 9.1 deg. Stimulus presentation blanked out the movie (with no interruption of the soundtrack) for 1 s, and occurred every 4 s on average (Figure 1). When testing adults (Experiment 1), three types of images were used: photographs of the sun; photographs of the moon, adjusted to match the mean luminance of the sun images; and phase-scrambled images of the sun that preserved mean luminance, power spectrum, and root mean square contrast (Olman et al., 2004). There were 13 images per category, all 10 × 10 cm (subtending 10 × 10 deg at 57 cm viewing distance). Each image was presented twice, over two sessions, in pseudorandomized order. For children (Experiment 2), only sun and phase-scrambled images of the sun were used, which yielded the strongest differences in pupillary response in adults. In addition, in separate sessions, full-screen white or black squares were shown for 1 s and 13 repetitions to estimate each child’s pupillary light/dark responses.

We verified that mean luminance (averaged across the image) was statistically indistinguishable between image categories, both in the set-up used with children (sun = 38.0 ± 3.4, phase-scrambled = 38.0 ± 3.4 cd/m^2^; two-sample *t*-test comparing luminance of the 13 sun pictures with the 13 phase-scrambled images: [*t*(25) = 0.00; *p* = 0.99; lgBF = –0.4] and in that used with adults [sun = 21.4 ± 1.7, phase-scrambled = 21.4 ± 1.7, moon = 21.4 ± 1.7 cd/m^2^; sun vs. phase-scrambled: *t*(25) = 0.00; *p* = 0.99; lgBF = –0.4; sun vs. moon: *t*(25) = 0.01; *p* = 0.99; lgBF = –0.4]. We also confirmed that the root mean square contrast of the sun and phase-scrambled images was strictly matched in both set-ups [children: sun = 30.3 ± 1.3, phase-scrambled = 30.2 ± 1.3: sun vs. phase-scrambled: *t*(25) = 0.02; *p* = 0.99; adults: sun = 14.6 ± 0.6, phase-scrambled = 14.5 ± 0.6: sun vs. phase-scrambled: *t*(25) = 0.01; *p* = 0.99; lgBF = –0.4].

Pupil diameter was monitored at 500 Hz with an EyeLink 1000 system (SR Research) with infrared camera mounted below the screen, recording from the left eye. Pupil measures were calibrated by an artificial 4-mm pupil placed at the approximate position of the participants’ eye. Synchronization between eye recordings and visual presentations was ensured by the EyeLink toolbox for MATLAB (Brainard, 1997).

### Analysis of Pupillometry and Eye-Tracking Data

Eye-tracking data were preprocessed using custom Matlab scripts that implemented the following steps:

1. Identification and removal of gross artifacts: removal of time-points with unrealistically small (< 0.1 mm, corresponding to blinks or other signal losses).
2. Identification and removal of finer artifacts: identification of samples where pupil size varied at unrealistically high speeds (> 10 mm per second, beyond the physiological range).
3. Removal of fragmented traces: identification of isolated segments (10 ms or less) that were separated from the rest of the trace (e.g., due to blinks or fast transients) and were therefore likely to consist of artifacts.
4. Down-sampling of data at 100 Hz, by averaging the retained time-points in non-overlapping 100 ms windows. If no retained sample was present in a window, that window was set to “NaN” (MATLAB code for “not a number”).

Pupil traces were transformed into changes from baseline by subtracting the average pupil diameter in the 1s preceding the pupillary response (i.e., from –800 to 200 ms after stimulus onset, the latter corresponding to the typical latency of the pupillary light response). After averaging all traces per subject and image type, we took the maximum dilation (for dark images) or the maximum constriction (for all other images) after the stimulus presentation to index the size of the response, which we submitted to statistical tests. Due to the preprocessing described above, trials with blinks or artifacts yielded traces with several missing values; we excluded these from our analyses by eliminating all trials for which no sample was available over the stimulus presentation window (mean ± s.e.m in adults: 1.7 ± 0.7%; autistic group: 15.5 ± 3.2%; comparison group: 5.3 ± 1.4%).

Gaze position from valid samples acquired during stimulus presentation were concatenated across trials and summarized by the Bivariate Confidence Ellipse area.

### Statistical Analysis

Statistics were computed with custom Matlab code and JASP (Team JASP, 2020). We used a repeated-measures approach, computing average per-participant responses and comparing them across stimulus types and (for children) across participant groups. Data from Experiment 1 were analyzed with a One-way ANOVA for repeated measures, with “stimulus category” as within-subject factor; subsequent paired *t*-tests tested pairwise differences between sun, moon and phase-scrambled images. Pupillometric results from Experiment 2 were analyzed with a mixed design ANOVA for repeated measures. This had a within-subject factor “stimulus category” (sun vs. phase-scrambled or bright vs. dark) and a between-subject factor “group” (autistic vs. comparison group). Results from both experiments were correlated with participants’ AQ scores using Pearson’s correlation coefficient. Gaze position statistics (Bivariate Confidence Ellipse area) were computed after pooling data across trials (irrespectively of stimulus category) and compared across groups using simple two-sample *t*-tests.

Each analysis was complemented with a Bayesian Repeated Measures ANOVA, which estimated Bayes Factors for each of the F-terms (Team JASP, 2020; van Doorn et al., 2020). Statistical significance was evaluated using both *p*-values and Bayes Factors. The Bayes Factor is the ratio of the likelihood of the two models H_1_/H_0_, where H_1_ assumes an effect (e.g., correlation between two variables or difference between two means) and H_0_ assumes no effect. By convention, the base 10 logarithm of the Bayes Factor lgBF > 0.5 is considered substantial evidence in favor of H_1_, and lgBF < –0.5 substantial evidence in favor of H_0_. Absolute values of lgBF greater than 1 are considered strong evidence, and greater than 2 decisive.

## Results

### Experiment 1: Pupillary Responses to Sun and Moon Pictures in Neurotypical Adults

**FIGURE 2:**
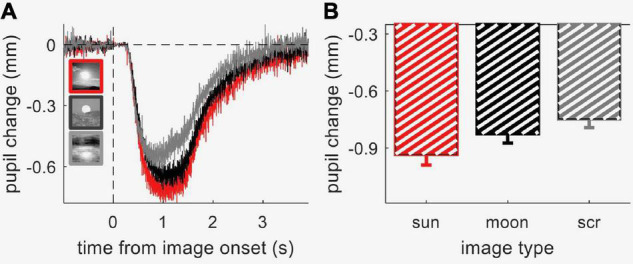
Results from Experiment 1 (neurotypical adults). **(A)** Timecourses of pupil constrictions (referenced to pre-stimulus baseline) evoked by the three image categories: sun, moon, and phase-scrambled sun images (“scrambled”); thin lines straddling the timecourse give s.e.m. at each timepoint. **(B)** Peak constriction for the three image categories, averaged across participants. Error-bars show s.e.m. across participants.

We measured pupil-size modulations with contextual information processing using a child-friendly paradigm, where the images (pictures of the sun, moon and phase-scrambled images) were embedded within an animated movie, which participants watched passively. We first validated this approach by measuring pupillary responses to the three image categories in 40 neurotypical adult participants. Figure 2A shows the average time course of pupil size (in mm) for each image category; Figure 2B shows the peak pupil constriction during the stimulus presentation window. Although all images were matched in luminance, pupil responses were clearly modulated by image category [one-way ANOVA for repeated measures, *F* = 14.5, *p* < 0.001, lgBF = 3.5]. *Post hoc t*-tests showed that the sun images evoked the strongest pupillary constriction, stronger than meaningless images obtained by phase-scrambling the sun images [*t*(37) = 5.2, *p* < 0.001, lgBF = 3.4] and stronger than moon pictures [*t*(37) = 2.5, *p* = 0.01, lgBF = 0.4]. Moon pictures also evoked stronger constriction than meaningless images [*t*(37) = 3.3, *p* = 0.002, lgBF = 1.2]. This pattern of results replicates previous findings (Binda et al., 2013b; Naber and Nakayama, 2013), indicating that the animation movie did not interfere with the processing of contextual information, which is presumably responsible for the modulation of the pupillary responses.

### Experiment 2: Pupillary Light Response in Children With and Without Autism

**FIGURE 3:**
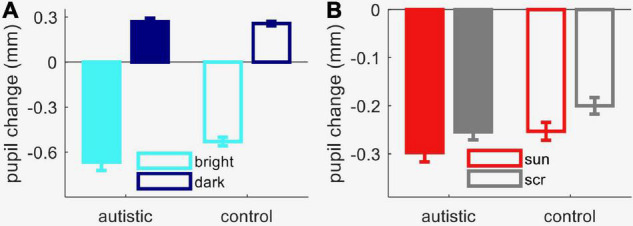
Results from Experiment 2 (autistic and comparison children). **(A)** Peak pupil response to luminance increments and decrements, averaged across observers in each group. **(B)** Peak pupil response to sun pictures and meaningless images (“scrambled”), averaged across observers in each group. In both panels, error bars show s.e.m. across participants.

As a preliminary step toward evaluating responses to the sun images in autistic participants and age- and IQ-matched controls, we measured the sensitivity of their pupillary system to simple light/dark stimuli (Figure 3A). We started by checking that the two groups were comparable in terms of gaze position distributions. The area of the Bivariate Confidence Ellipse of gaze position samples during the stimulus presentation for the autistic group 14.1 ± 4.4 deg^2^, and for the comparison group 9.1 ± 2.3 deg^2^, not significantly different: [*t*(39) = 1.1, *p* = 0.28, lgBF = –0.3]. This ensured that any variations in pupil reactivity cannot be explained by group differences in eye movements.

We next studied pupil reactivity with a mixed-design ANOVA, which revealed a significant interaction between the within-subject factor stimulus category (bright or dark screen) and the between-subject factor autism diagnosis [interaction term: *F*(1, 39) = 6.1, *p* = 0.018, lgBF = 0.7]. *Post hoc* tests showed that the autistic group had a marginally stronger pupillary response to light than the comparison group [two-sample *t*-test: *t*(39) = 2.04, *p* = 0.04, lgBF = 0.2]. However, the amplitude of the pupillary dark response was indistinguishable between groups [two-sample *t*-test: *t*(39) = 0.3, *p* = 0.76, lgBF = –0.5], as was the overall shape of both responses, with no apparent difference in dynamics or latency (not shown). Autistic participants also had slightly more dilated resting pupil diameter measured in the pre-stimulus interval [mean ± standard error of the mean, autistic group: 4.1 ± 0.14 mm; comparison group: 3.8 ± 0.1 mm; *t*(39) = 1.8, *p* = 0.08, lgBF = 0.02]. It is possible that this small difference in resting diameter contributes to the enhanced pupillary light response, since a more dilated resting diameter may leave more room for a light evoked constriction.

### Pupillary Responses to Contextual Images in Children With and Without Autism

Finally, we probed contextual information processing in our child participants by focusing on responses to the sun and phase-scrambled images, those most distinct in the adult data.

**FIGURE 4:**
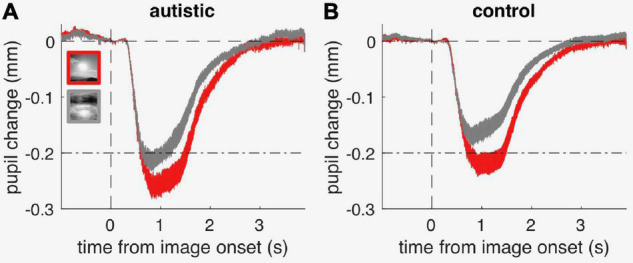
Timecourses of pupillary constriction (referenced to pre-stimulus baseline) evoked by sun and phase-scrambled sun images (“scrambled”), in the group of autistic children **(A)** and in the comparison group **(B)**; thin lines straddling the timecourse give s.e.m. at each timepoint. The dash-dot lines serve as reference to highlight the difference in response amplitude across panels.

Figures 4A,B shows the average time-course of pupil responses, separately for participants with and without autism. Although pupil constriction responses were generally stronger in the autistic than the comparison group (in line with the trend for an enhanced pupillary response to light seen in Figure 3A), both groups clearly showed different pupil responses depending on image category, like the neurotypical adults. Figure 3B shows average pupil constriction during the stimulus presentation window, which were analyzed with a mixed-design ANOVA with factors: stimulus category (sun, phase-scrambled: within-subjects) and diagnosis (autism and non-autism, between-subjects). This confirmed that pictures of the sun systematically evoked a stronger constriction than the meaningless phase-scrambled images [main effect of image category: *F*(1, 39) = 92.5, *p* < 0.001, lgBF = 8.7], and also supported the marginally stronger pupillary constriction in the autistic than comparison group [main effect of diagnosis: *F*(1, 39) = 3.8, *p* = 0.058, lgBF = 0.0]. However, the effect of image category was indistinguishable between the autistic and comparison groups [no image by diagnosis interaction: *F*(1, 39) = 0.1, *p* = 0.79, lgBF = –0.0], as confirmed by the non-significant difference between the two [*t*(39) = 0.3, *p* = 0.79, lgBF = –0.5].

Taking into account the enhanced pupil responsivity in the autistic group did not change the pattern of results: after normalizing each individual response by their pupillary light response, the amplitude of the response to the sun pictures was indistinguishable between groups [*t*(39) = 0.5; *p* = 0.69; lgBF = –0.5].

We also checked and confirmed that gaze position did not differ between groups [BCE area in the autistic group: 5.4 ± 1.7 deg^2^; comparison group: 2.3 ± 0.6 deg^2^; *t*(39) = 1.8, *p* = 0.07, lgBF = 0.1).

As children in the comparison group had heterogeneous diagnoses, we also checked that pupillary responses did not differ across sub-groups (ADHD, LD, DLD, BD) with a one-way ANOVA on pupillary responses to the sun pictures [*F*(4, 36) = 1.0, *p* = 0.4, lgBF = –0.4]. Levene’s test also confirmed that pupillary responses to the sun and phase-scrambled pictures were similarly variable across groups [*F*(1) = 0.0, *p* = 0.89; *F*(1) = 0.5, *p* = 0.46 respectively].

### Contextual Pupil Response and Autistic Traits

**FIGURE 5:**
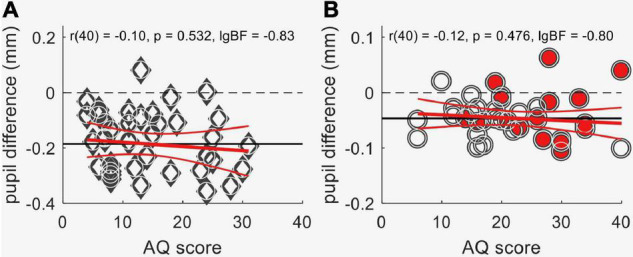
Lack of association between autistic-like traits, as measured by the Autism-spectrum Quotient questionnaire (AQ score), and the difference in pupil response evoked by the sun images and the meaningless control images obtained by phase scrambling. **(A)** Data from neurotypical adults. **(B)** Data from children, from both the autistic (filled symbols) and the comparison groups (empty circles). The horizontal continuous black lines show the mean, and the red lines the best-fit linear regression with its 95% confidence interval. Text insets give the Pearson’s correlation (with sample size) and associated *p*-value and log Bayes Factor.

Previous reports of reliable associations between pupillometry results and inter-individual differences in perceptual styles (Turi et al., 2018; Pome et al., 2020; Tortelli et al., 2020, 2021) motivated us to investigate the relationship between the effects of contextual information on pupillary responses and autistic-like traits measured by the Autism-Spectrum Quotient (Baron-Cohen et al., 2001). However, we found no association between AQ and the pupil difference for sun pictures and phase-scrambled images, neither in neurotypical adults [Figure 5A: *r*(38) = 0.1; *p* = 0.45; lgBF = –0.8] nor in the groups of children (Figure 5B: *r*(40) = –0.03; *p* = 0.84; lgBF = –0.9].

## Discussion

We measured pupil size changes in response to images of the sun, moon and phase-scrambled images, all matched in average luminance. Previous studies showed that these images evoke a different pupillary constriction response in adults, despite being matched in luminance, indicative of an effect of contextual information on pupillary responses. Here we replicated the findings of Binda et al. (2013b) in a group of neurotypical adults (Experiment 1), using a novel, child-friendly setup, without requiring participants to respond: the high-luminance context implied by the sun image caused higher pupillary constriction, even when embedded within an attention-grabbing animation movie. We then measured the effect in two groups of 6–15 year-old children (Experiment 2), one group with autism diagnosis, the other with unrelated development disorders. Both groups showed similarly strong contextual effects, with pupils constricting significantly more for the sun than for the phase-scrambled control image: there was no measurable difference between the two groups.

The heterogeneity of our comparison group might in principle have inflated the variance of the measurements, decreasing our ability to measure group differences. However, our results speak against this possibility. First, we find that the two groups are well matched for inter-individual variability (a requirement for most statistical comparisons and one that would likely have not been met with a control group of neurotypical controls). Second, we tested for differences within our control group depending on the diagnostic subcategory and found none. Third, we found significant pupil modulations in response to sun vs. phase-scrambled pictures in both groups, indicating that our test was sensitive to relatively subtle pupil modulations.

We chose to study this clinical comparison group, rather than a group of neurotypical participants, to mitigate the risk that non-specific developmental deficits (including attentional deficits, sensory-motor anomalies) would introduce spurious differences between groups. This is important if the final goal is to establish quantitative indices of perception (e.g., through pupillometry), which could help clinical evaluation and diagnosis by differentiating autism from other neurodevelopmental disorders, despite symptoms in partially overlapping areas. Also, we chose to study participants with relatively high cognitive profile; this implies that our conclusions may not be relevant to the component of the autistic population that is characterized by cognitive impairment—a limitation that may be addressed in future studies.

It is now established that pupil diameter is sensitive to top-down modulation, implying that it is modulated by cortical pathways other than the subcortical PLR system (Binda and Murray, 2015; Ebitz and Moore, 2018). A recent experiment using continuous flash suppression (Sperandio et al., 2018) demonstrated that extra-retinal pupillary modulation requires visual awareness: the pupillary response to the sun and phase-scrambled images differed only when participants were aware of the images, not when the images were successfully suppressed from awareness. This is clear evidence that the pupil modulation evoked by pictures of the sun reflects high-level perceptual processing and the contents of conscious perception. Our results suggest that this form of perceptual processing, albeit relatively high-level, is not affected in autistic individuals. This negative finding is in line with a recent study that used pupillometry to compare perceptual processing in autistic individuals and controls and failed to reveal systematic differences in pupillary constriction to illusory bright stimuli (Laeng et al., 2018).

On the other hand, the literature reports multiple instances of differences in basic pupillary responses to light or dark in autism, including reports of enhanced pupillary responses to light in autistic individuals compared with controls (Nystrom et al., 2015, 2018); our findings are in line with this pattern (Figure 3A) and might be linked to hypersensitivity phenomena that are often associated with autism (Williams, 1994; Robertson and Baron-Cohen, 2017). However, the literature presents discordant findings, with some studies reporting no differences with autism, or reporting differences in latency but not in amplitude (Dinalankara et al., 2017; Lynch et al., 2018); some studies even show the opposite pattern (Fan et al., 2009; Kercher et al., 2020). For example, Fan et al. (2009) reported that pupils of autistic children took longer to respond to short (0.1 s) light stimuli, and constricted less and more slowly than those with typical development. We also found a marginally larger pre-stimulus pupil diameter in the autistic group, in line with other studies (Anderson et al., 2006; Anderson and Colombo, 2009; Kercher et al., 2020). Also in this case, however, the literature includes conflicting reports, with some finding a weak (Martineau et al., 2011) or non-existent (Fan et al., 2009; Nystrom et al., 2015, 2018) steady-state pupil size difference between autistic individuals and controls.

These discrepancies suggest that, although pupillary light responses and steady-state pupil diameter may be the easiest pupillometry parameters to estimate, they are not necessarily the most informative, being unable to systematically differentiate autistic individuals from controls (at least not consistently across the varying testing conditions in different studies). Measuring pupil size modulations related to more complex aspects of visual processing may provide a more informative index—one that may be able to track the contents of perception or cognition. The negative findings in the present study, together with a previous report (Laeng et al., 2018), should not discourage this pursuit but may rather testify to its selectivity.

Our research was motivated in part by the recent flurry of Bayesian theories of autistic perception (Pellicano and Burr, 2012; Friston et al., 2013; van Boxtel and Lu, 2013; Lawson et al., 2014; Sinha et al., 2014; Van de Cruys et al., 2014; Rosenberg et al., 2015; Palmer et al., 2017)., which suggest that the perception of autistic individuals may be more “data driven,” and less susceptible to contextual information. In the Bayesian context this would mean weaker *priors.* This may predict that the contextual inference of the bright sun should be weaker and drive the pupillary response less. However, since the original suggestion by Pellicano and Burr (2012), research suggests that autistic perception is not so much characterized by intrinsically weak *priors*, but by less flexible priors (Lawson et al., 2017; Shi et al., 2022). In our paradigm there was little unpredictability, with images that were always sun-like, or scrambled. Perhaps, however, paradigms of this sort could be adapted to test objectively the suggestion that Bayesian *priors* in autism are less flexible.

## Conclusion

Previous studies have stressed the importance of minimizing the effects of decision biases when assessing inter-individual differences in perceptual experience—such as those that may emerge between individuals with and without autism. The child-friendly no-report paradigm introduced here might serve this purpose in future studies. Specifically, it may help determine whether atypical perception (as previously reported in autistic persons for visual illusions or local-global hierarchical images) reflect real perceptual differences between autistic and neurotypical individuals. Also, it may be useful to identify which (if any) of these functions is selectively altered in autism.

## Data Availability Statement

The datasets presented in this study can be found in online repositories. The names of the repository/repositories and accession number(s) can be found below: 10.5281/zenodo.4608371.

## Ethics Statement

The studies involving human participants were reviewed and approved by the Comitato Etico Pediatrico Regionale—Azienda Ospedaliero-Universitaria Meyer—Firenze (FI). Written informed consent to participate in this study was provided by the participants’ legal guardian/next of kin.

## Author Contributions

CT and AP conducted the data acquisition. RI and MT did the selection of participants. All authors contributed to the conceptualization of the research, and the design, analysis and interpretation of the results, read and approved the final manuscript.

## Conflict of Interest

The authors declare that the research was conducted in the absence of any commercial or financial relationships that could be construed as a potential conflict of interest.

## Publisher’s Note

All claims expressed in this article are solely those of the authors and do not necessarily represent those of their affiliated organizations, or those of the publisher, the editors and the reviewers. Any product that may be evaluated in this article, or claim that may be made by its manufacturer, is not guaranteed or endorsed by the publisher.
